# Effect of ZnO Nanoparticles on Growth and Biochemical Responses of Wheat and Maize

**DOI:** 10.3390/plants10122556

**Published:** 2021-11-23

**Authors:** Akansha Srivastav, Deepak Ganjewala, Rakesh Kumar Singhal, Vishnu D. Rajput, Tatiana Minkina, Marina Voloshina, Sudhakar Srivastava, Manoj Shrivastava

**Affiliations:** 1 Centre for Environment Science and Climate Resilient Agriculture, ICAR-Indian Agricultural Research Institute, New Delhi 110012, India; akansha9110@gmail.com; 2 Amity Institute of Biotechnology, Amity University, Noida 201303, India; dganjewala@amity.edu; 3 Analytical Chemistry Division, Bhabha Atomic Research Centre, Mumbai 400085, India; rakeshsinghal65@gmail.com; 4 Academy of Biology and Biotechnology, Southern Federal University, 344090 Rostov-on-Don, Russia; rajput.vishnu@gmail.com (V.D.R.); tminkina@mail.ru (T.M.); ph.rox.anderson@gmail.com (M.V.); 5 Institute of Environment and Sustainable Development, Banaras Hindu University, Varanasi 221005, India; sudhakar.iesd@bhu.ac.in

**Keywords:** antioxidant enzymes, α-amylase, dehydrogenase, lipid peroxidation, zinc accumulation

## Abstract

Zinc is an essential element that is also renowned for widespread contamination and toxicity at high concentrations. The present study was carried out to analyze the responses induced by lower, as well as higher, doses of zinc (0–200 mg/L), in the form of zinc oxide nanoparticles (ZnO NPs) in wheat and maize, for a period of 21 days. Accumulation of zinc increases with increasing Zn doses in both wheat and maize, with higher doses being in wheat (121 mg/kg in root and 66 mg/kg in shoot) than in maize (95 mg/kg in root and 48 mg/kg in shoot). The activity of alpha-amylase showed increase, while that of dehydrogenase decline, in response to ZnO NPs. The length and biomass of plants and photosynthetic pigments increased slightly upon ZnO NPs supply. Malondialdehyde content showed a progressive increase in root and shoot of both plants. However, in response, antioxidant enzymes (superoxide dismutase, ascorbate peroxidase, guaiacol peroxidase, and catalase) showed increase up to lower concentrations (100 mg/L) of ZnO NPs but decline variably at higher levels (150–200 mg/L) in wheat and maize. The results suggest that lower supply of ZnO NPs (100 mg/L) could be stimulatory to the growth of plants and can be recommended as a Zn fertilizer source for crop production.

## 1. Introduction

Nanotechnology is an emerging field of science, whose potential is seen in almost every facet of life. The field of nanotechnology uses various engineered nanoparticles (NPs) and encompasses various fields of science under one umbrella, including biology, physics, chemistry, and engineering. The areas starting from drug delivery to wastewater cleanup, soil remediation, and controlled fertilizers come under the purview of nanotechnology. The use of a variety of NPs is increasing day by day in these fields, including agriculture, where NPs have found uses as fertilizers and stress-counteractive agents [1]. An engineered nanoparticle refers that particle having their size less than 100 nm, with at least one dimension, e.g., titanium dioxide (TiO_2_), zinc oxide (ZnO), etc. However, as the use of NPs is increasing, the chances of their accidental spillage to unwanted locations, such as ponds or groundwater, are also increasing [2].

Zinc (Zn) is an essential micronutrient for both plants and animals owing to its involvement in several enzyme activities and metabolic functions. Zinc deficiency in plants leads to poor yield levels, while, in humans, it malnutrition and several ailments [3,4]. However, it also poses serious threats to soil and water ecosystem, if present in quantities more than permissible limits. The critical soil value of Zn is 70 to 400 mg/kg [5]. There are several sources through which Zn is released into the environment, such as mining, smelting operations, and agriculture. The excess of Zn in soil and water reaches plants and ultimately biomagnifies through the food chain to accumulate in higher then optimal levels in humans [4]. The presence of Zn in the system can function as a stress that causes physiological and biochemical changes that leads to plant growth inhibition [6].

The zinc oxide nanoparticle (ZnO NP) is a commonly used metal oxide ENPs finding application as sunscreens and cosmetics, biosensors, and in solar cells, etc. [7]. Presently, many kinds of metal oxide NPs have been applied in agriculture, specifically in fertilization and plant protection in abiotic and biotic stress conditions [8,9]. The effects of NPs on plant germination, growth, and biochemical responses have been studied widely, in the recent past, with the goal of sustaining agricultural production. Nanoparticles are released through deliberate application and accidentally, due to increasing use in consumer products and other fields, reaching to aquatic, terrestrial, and atmospheric environments. The unique properties of NPs and their inadvertent contamination could potentially lead to unexpected health or environmental hazards [10]. Therefore, organisms, such as algae, plants, and fungi, are expected to be affected, as a result of their exposure to NPs. Zinc oxide nanoparticles solubility decreases if the soil pH increases, and that is the main reason ZnO NPs are less available to plants [11]. Transport of these NPs into different plant parts may be limited, due to the presence of different kind of barriers found in roots [12]. However, increased production and release of ZnO NPs may cause adverse effects on terrestrial and aquatic ecosystems.

Wheat is widely used crop, as it is consumed by 50% of the population as a staple diet, because wheat contains dietary fibers, carbohydrates, and more vegetable proteins, which are required for human nutrition [13]. Maize is used as a third most important crop, after rice and wheat. It is not only used for human and animal feed, but it is widely used in the corn starch, baby corn, and corn oil industries, as well [14]. The aim of this study was to access the impact of ZnO NPs on crop plants and find its optimum dose. The hypothesis was that ZnO NPs would play a beneficial role at lower doses, while, at higher doses, the increasing accumulation of Zn might lead to growth reduction and stress to plants. Plants are the essential base components of the ecosystem and give the initial energy for primary consumers of a food chain; therefore, wheat and maize were used as model plants in this study.

## 2. Materials and Methods

### 2.1. Expterimental Setup, Seed Germination, and Enzyme Assay Analyses

Wheat (*Triticum aestivum*) and maize (*Zea mays*) seeds were sterilized in a 10% (*v/v*) NaClO_4_ solution for 10 min and then thoroughly rinsed with sterilized deionized water. After rinsing, seeds were soaked in ZnO NPs suspension at various concentrations (0, 50, 100, 150 and 200 mg/L). Then seeds were placed onto glass Petri dishes, with filter papers at the bottom (ten seeds per dish for wheat and five seeds per dish for maize); then, five mL deionized water (control) or different concentrations of NPs suspensions were added to the dishes. The Petri dishes, after sealing with tape, were kept in an incubator at 25 °C. After 5 days, the germination was halted, and the seedling root length was measured by a millimetre ruler.

The germinated seeds were used for enzyme assays. The activity of alpha-amylase was assayed by following the method [15]. One gram of treated and control seedlings were homogenized in 0.1 M sodium acetate buffer (pH 4.8) containing 10 mM NaCl and centrifuged. Enzyme reaction mixture contained 2.0 mL of 0.1 M sodium acetate buffer (pH 4.7), 0.5 mL of 1% starch, and 0.5 mL enzyme extract in total 3 mL volume. The reaction mixture was incubated for 10 min at 37 °C; then, 2 mL of dinitrosalicylic acid (DNS) was added and reaction was heated in a water bath at 100 °C for 10 min. Absorbance was taken at 510 nm. Total dehydrogenase activity for germinated seedlings was determined by homogenizing seeds with 0.1 M sodium phosphate buffer, pH 7.2, containing 1.5% (*w/v*) 2,3,5-triphenyltetrazolium chloride. Samples were incubated at 25 °C for 24 h; then, 10 mL methanol was added. The absorbance was taken at 510 nm [16].

**Figure 1:**
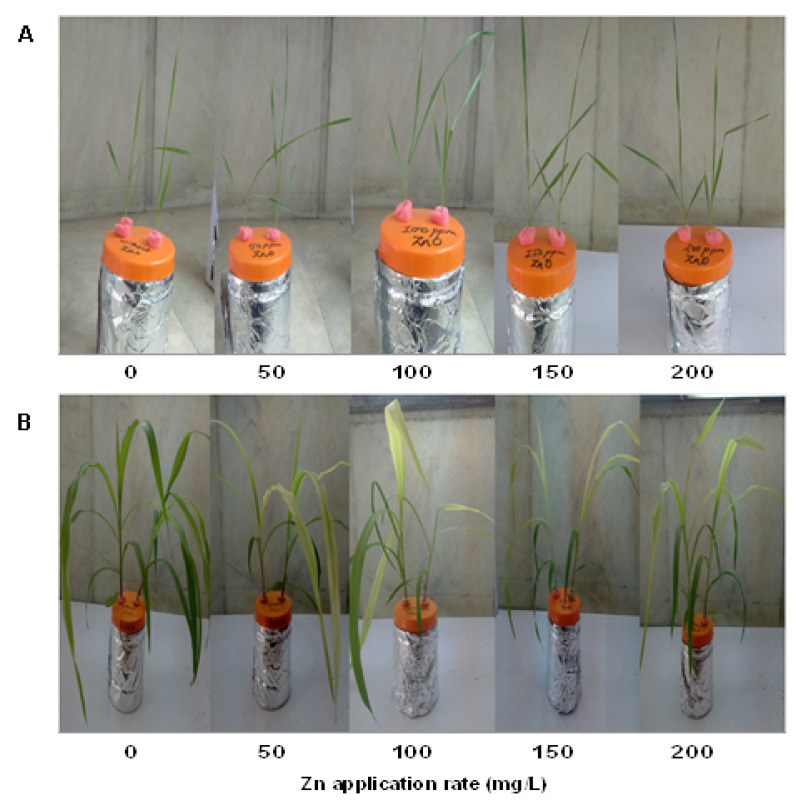
Representative images of plants of wheat (**A**) and maize (**B**) treated with different doses of zinc oxide nanoparticle.

Germinated seeds were grown for 21 days in hydroponic filled with 1/4th strength Hoagland’s solution (Figure 1). All the treatments were set in tetraplicate and each replicate contained four plants of equal size. Plants were treated with different concentrations of Zn, by using ZnO NPs, maintained in 1/4th strength Hoagland’s solution. After 21 days, plants were harvested and washed with double distilled water and used for further studies.

### 2.2. Zinc Quantification in Plant Tissues

Harvested plants were washed with distilled water and oven dried at 60 °C. Sample preparation for Zn estimation was performed by digesting the plant material in HClO_4_: HNO_3_ (1:3, *v/v*) at 90 °C and then diluting the samples with distilled water. Zinc contents in digested samples were determined on a flame atomic absorption spectrophotometer (AA-7000, SHIMADZU).

### 2.3. Plant Biomass and Photosynthetic Pigments Measurement

Biomass of plants was measured on fresh weight and dry weight basis. For photosynthetic pigments, 100 mg leaves of treated and untreated plants were extracted in 10 mL of chilled acetone:ethanol (1:1, *v/v*). This extract was centrifuged at 10,000× *g* for 10 min, and the supernatant was collected. After centrifugation, absorbance of the supernatant was taken at 663, 645, 480, and 510 nm. The total chlorophyll content, as well as chlorophyll a, chlorophyll b, and carotenoid content, was calculated in mg, per g of fresh weight, using the following formulae [17,18]:

Total chlorophyll (mg/g fw) = {[20.2(A_645_) + 8.02 (A_663_)] × V} ÷ (1000 × W)

Chlorophyll a (mg/g fw) = {[12.7(A_663_)–2.63(A_645_)] × V} ÷ (1000 × W)

Chlorophyll b (mg/g fw) = {[22.9(A_645_)–4.68(A_663_)] × V} ÷ (1000 × W)

Carotenoid (mg/g fw) = {[7.6 (A_480_–2.63(A_645_)] × V} ÷ (1000 × W)

where,

A_663_, A_645_, A_510_ and A_480_ = Absorbance at 663, 645,510 and 480 nm
V = Final extract volume (mL)
W = Weight of the sample (g).

### 2.4. Lipid Peroxidation and Assay of Antioxidant Enzymes

Lipid peroxidation of plant materials was determined by the estimation of the malondialdehyde (MDA) content, following Heath and Packer [19]. The MDA content was calculated, according to its molar extinction coefficient of 155 mM^−1^ cm^−1^. For the enzyme assay, roots and shoots (250 mg each) of the control and treated plants were crushed separately in a pre-chilled mortar and pestle, using liquid nitrogen (N2), until a fine powder was obtained. The powder was then suspended in 3 mL of sodium phosphate buffer (0.1 M, pH 6.2) and centrifuged at 8000× *g* for 15 min at 4 °C. The supernatant was used to estimate the activities of antioxidative enzymes, namely superoxide dismutase (SOD), ascorbate peroxidase (APX), guaiacol peroxidase (GPX), and catalase (CAT), by following methods detailed in Singh et al. [20]. SOD activity was determined by photochemical reduction of nitrobluetetrazolium (NBT). For APX activity, the reaction mixture containing phosphate buffer (25 mM, pH 7.0), EDTA (0.1 mM), APX (0.25 mM), H_2_O_2_ (1.0 mM), and enzyme extract was used. Reduction in absorbance was measured at 290 nm for 60 s. GPX activity was determined in terms of oxidation of guaiacol to tetraguaiacol and change in absorbance was monitored at 470 nm. CAT activity was assessed, with respect to H_2_O_2_ dissociation, and the decrease in absorbance was monitored at 240 nm. The activity of all enzymes is expressed as U g^−1^ FW [20].

### 2.5. Characterization of Used Nanoparticles

**Figure 2:**
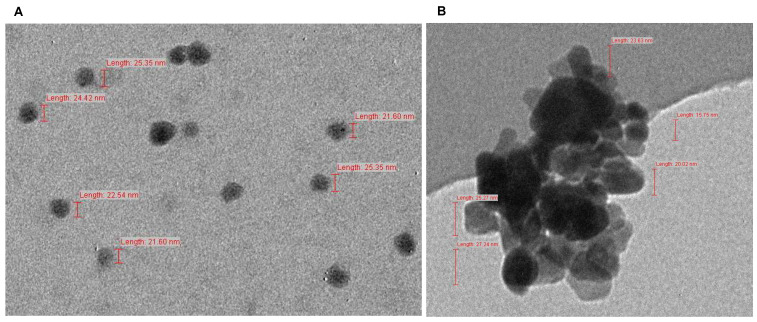
Individual primary ZnO nanoparticles at magnification of 100,000× (**A**) and aggregate-ed ZnO nanoparticles at magnification of 300,000× (**B**), as seen under transmission electron microscope.

ZnO nanoparticles were purchased from Sigma-aldrich, Saint Louis, MO 63103, USA, with a purity of 97.0% and average particle size of ≤50 nm. Further, the size distribution of ZnO NPs was evaluated by transmission electron microscopy (TEM) (Model JEM1011) with magnifications between 100,000× and 320,000× and an acceleration voltage of 100 kV coupled to a CCD camera, allowing digital images acquisition for the particles size and morphology determinations. Two mg ZnO NP was mixed in 6 mL of distilled water, and then the mixture was stirred and sonicated for about 4 h in an ultra sonicator with frequency of 35 KHz. A drop of this suspension was deposited on copper TEM grids (100 mesh covered with carbon film), dried within a laminar flow in a fume cupboard, and observed under TEM. The obtained images are mostly composed of individual primary particles (Figure 2A) and aggregated ZnO NPs (Figure 2B), which are mainly in spherical in shape, with a size between 15 and 40 nm (Figure 2A,B).

### 2.6. Statistical Analysis

Experiment was conducted in a completely randomized block design, and each treatment was performed in four replicates. Statistical analyses were performed using MS Excel, and the statistical significance was determined by the *p* value ≤ 0.05 (or ≤0.01) in a Student’s *t* test [21].

## 3. Results and Discussion

### 3.1. Seed Germination and Enzyme Activities

**Figure 3:**
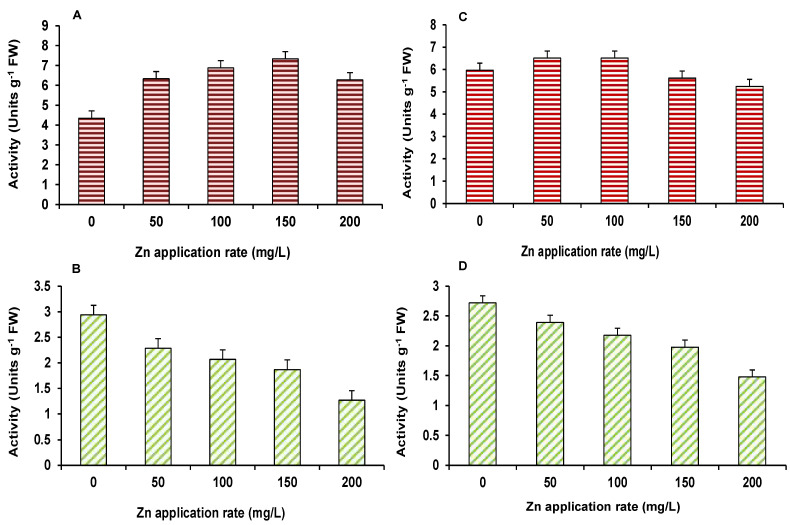
Effect of ZnO NPs treatment on germination related alpha-amylase (**A**,**C**) and dehydrogenase (**B**,**D**) activities of maize (**A**,**B**) and wheat (**C**,**D**). Values are means of four replicates. Error bars indicate the least significant value (LSD) at *p* ≤ 0.05 among the treatments.

Result on α–amylase and dehydrogenase activities, in the germinated seedlings of maize and wheat, are presented in Figure 3A–D. All treatments led to 100% germination of seeds. The activity of α–amylase was increased, as the concentration of ZnO NPs increased in wheat and maize seedlings; however, at 150 and 200 mg/L, α–amylase activity decreased in wheat plants, as compared to the control. The dehydrogenase activity showed a consistently declining trend, in both wheat and maize, in response to ZnO NPs, as compared to the control (Figure 3). Seed germination and early seedling establishment constitute the most important phase of plant development. Successful early growth of seedlings strengthens them and enables them to tolerate stresses effectively [22]. In this study, seeds showed 100% germination in all treatments, suggesting no negative impact of ZnO NPs. This is validated by earlier findings of Lin and Xing [23], who reported seed germination was affected by ZnO NPs in corn only at 2000 mg/L level. Amylase is an enzyme found in the germinating seeds, while dehydrogenase is important, as it catalyzes the stored products in anaerobic phase of seed germination. This will lead to the release of energy in the early growth of seeds in germination [24]. Amylase activity is affected by many factors, which include temperature, enzyme concentration, pH, and substrate concentrations.

### 3.2. Accumulation of Zinc in Different Plant Parts and Effect on Growth of Plants

**Figure 4:**
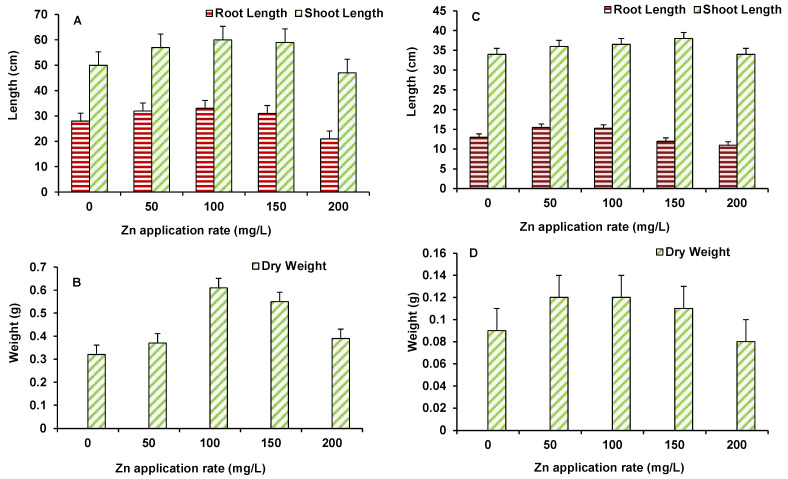
Effect of ZnO NPs treatment on root and shoot length (**A**,**C**), and dry weight (**B**,**D**) of maize (**A**,**B**) and wheat (**C**,**D**). Values are means of four replicates. Error bars indicate the least significant value (LSD) at *p* ≤ 0.05 among the treatments.

Data on root and shoot length and dry biomass of maize and wheat are presented in Figure 4A–D. For maize, root and shoot length and biomass increased up to 100 mg/L ZnO NPs treated plants (Figure 4A,B). However, higher ZnO NPs level induced some decline in growth parameters with the root and shoot length being lower than the control, only at 200 mg/L (Figure 4A,B). Similarly, in wheat, the length and biomass of plants showed higher control values, up to 150 mg/L ZnO NPs. At the maximum dose of 200 mg/L ZnO NPs, both length and biomass of plants were found to be lower than the control (Figure 4C,D).

**Figure 5:**
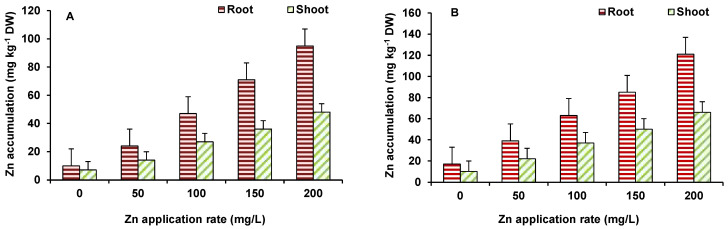
Effect of ZnO NP treatment on Zn accumulation in maize (**A**) and wheat (**B**). Values are means of four replicates. Error bars indicate the least significant value (LSD) at *p* ≤ 0.05 among the treatments.

Accumulation of Zn in roots was higher, as compared to shoots in both wheat and maize (Figure 5A,B). The maximum Zn accumulation was observed at 200 mg/L ZnO NPs treated wheat (121 ppm in roots and 66 ppm in shoots) and maize (95 ppm in roots and 37 ppm in shoots) plants. Initially up to 100 mg/L ZnO NPs, enhanced length and biomass of plants. Zinc is an essential element for plants and has a role in protein synthesis and in activity of several enzymes, as well as in membrane integrity, metabolic reactions, water uptake and transport, and gene expressions [25]. Hence, an increase in Zn level was beneficial for the plants, leading to improvement in growth. However, toxicity occurred at higher ZnO NPs treatment (150–200 mg/L) level to some extent that might be due to perturbed homeostasis of Zn and indirect effects on other element uptake and mutual elemental interactions. Zinc application, in different forms, has been found to important protection against a number of abiotic stresses and improve growth of plants. However, only a few studies have demonstrated the effect of ZnO NPs on plant growth and productivity [23,26] and observed that ZnO NPs affect the growth and yield of plants, and Zn accumulates in various tissues, including grains/produce. ZnO-NPs shows enhanced plant growth, up to a certain concentration [27], where Zn^2+^ has been provided as micronutrient [28].

### 3.3. Effect of Photosynthetic Pigments

**Figure 6:**
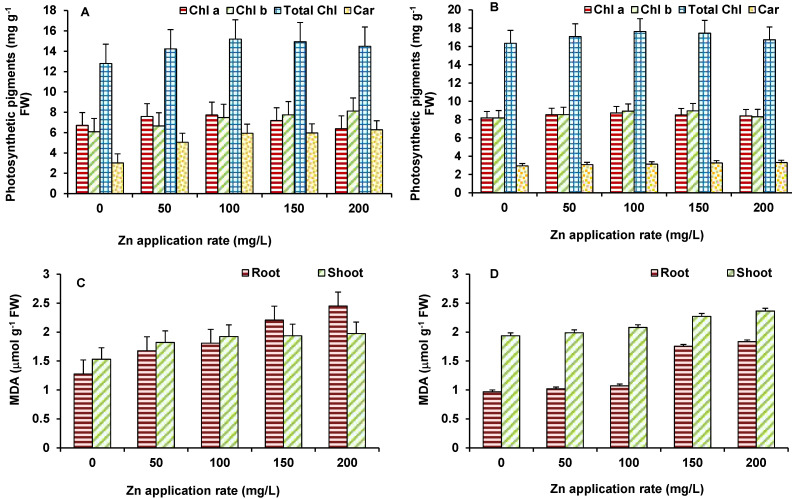
Effect of ZnO NPs treatment on photosynthetic pigments and MDA content of maize (**A**,**C**) and wheat (**B**,**D**). Values are means of four replicates. Error bars indicate the least significant value (LSD) at *p* ≤ 0.05 among the treatments.

ZnO NPs exhibited similar responses on photosynthetic pigments (Chl a, Chl b, total Chl, and carotenoid) upon Zn exposure in maize and wheat plants. The level of Chl a, Chl b, and total Chl was increased as the concentration of ZnO NPs increased (Figure 6A,B). The maximum increase in Chl a (19% in maize and 25% in wheat), Chl b (30% in maize and 24% in wheat), and total Chl (50% in maize and 50% in wheat) was observed at 200 mg/L ZnO NPs. Similar to chlorophylls, carotenoids were also increased as the concentration of Zn increased for both the plants. The maximum increase in carotenoids in maize and wheat occurred at 200 mg/L ZnO NPs (Figure 6A,B). Photosynthesis is one of the primary reactions affected by stresses and by any disturbance to metabolism of the plants. Therefore, Zn accumulation was found to be positive stimulator of chlorophyll and carotenoid synthesis that would have led to improved photosynthetic efficiency of plants evident in increased biomass. Further, Zn is crucial for the activity of carbonic anhydrase that mediate hydration of CO_2_ to bicarbonate for delivery to the chloroplasts [29] Carbonic anhydrase also plays role in stomata regulation [30]. Thus, optimum Zn supply helps plants improve photosynthesis.

### 3.4. Effect on Lipid Peroxidation and Antioxidant Enzymes

In order to evaluate the membrane damage imposed by ZnO NPs, MDA content (Figure 6C,D) was measured to analyze lipid peroxidation. MDA increased gradually with increasing concentration of Zn in both the plants. The maximum increase in MDA in shoots (12% in maize and 18% in wheat) and roots (47% in both maize and in wheat) was observed at 200 mg/L ZnO NPs (Figure 6C,D).

**Figure 7:**
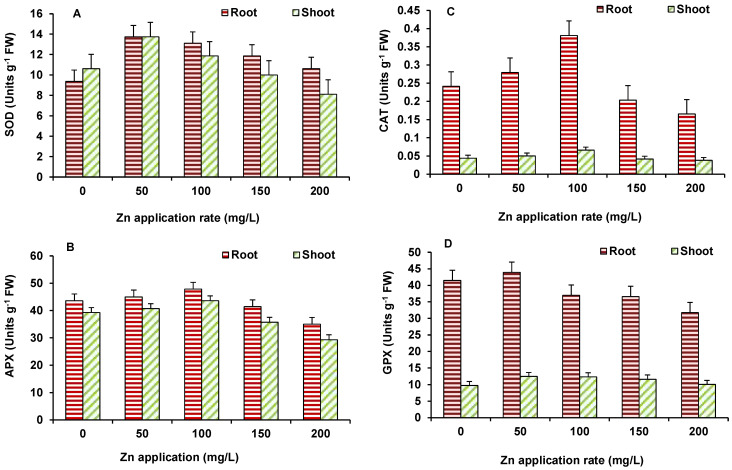
Effect of ZnO NP treatment on activity of SOD (**A**), APX (**B**), CAT (**C**), and GPX (**D**) of maize. Values are means of four replicates. Error bars indicate the least significant value (LSD) at *p* ≤ 0.05 among the treatments.

In this study antioxidant enzymes showed varying response with increase in ZnO NPs concentrations. The used NPs have the capability to stimulate reactive oxygen species (ROS) generation in the system, which leads to cell death when the antioxidative capacity of the cell increases [31]. The activity of SOD in maize (Figure 7A) increased at 50 mg/L ZnO NPs but decreased afterwards; however, it was higher than the control at all ZnO NPs treated treatments. The activity of APX in both root and shoot of maize was higher than the control up to 100 mg/L ZnO NPs but declined at higher ZnO NPs treatment, as compared to the control (Figure 7B). A similar response was seen for CAT activity (Figure 7C). GPX activity (Figure 7D) showed decline, in comparison to the control, at 100 mg/L onwards in roots and shoots.

**Figure 8:**
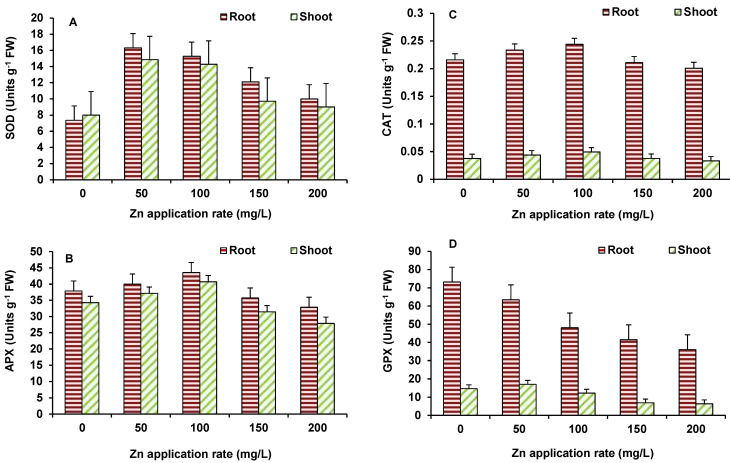
Effect of ZnO NPs treatment on activity of SOD (**A**), APX (**B**), CAT (**C**), and GPX (**D**) of wheat. Values are means of four replicates. Error bars indicate the least significant value (LSD) at *p* ≤ 0.05 among the treatments.

In case of wheat also, the activity of SOD (Figure 8A), APX (Figure 8B) and CAT (Figure 8C) showed significant increase mostly up to 100 mg/L ZnO NPs and then after decline occurred in the activity. However, significant decline, in comparison to the control, occurred only in APX at 200 mg/L ZnO NPs treated treatments. GPX activity (Figure 8D) depicted decline at all concentrations in shoot and 100 mg/L ZnO onwards in roots, as compared to the control.

Zinc is well-known for its role as a cofactor of SOD and, therefore, it performs as an antioxidant and helps plants in quenching of ROS [32]. In the present study, an increase in Zn levels upon ZnO NPs supply, the activity of various antioxidant enzymes mostly depicted an increase, particularly up to 100 mg/L ZnO NPs. This would have helped plants in the regulation of ROS and assisted in achieving improved growth. At higher ZnO NPs levels, although Zn accumulation increased, it presumably overburdened plants and disturbed Zn homeostasis. This resulted in higher stress that was evident in increased MDA levels and decreasing trend of antioxidant enzymes and growth, as well. Hence, lower concentrations of ZnO NPs were stimulatory to plants. In an earlier study by Faizan et al. [33], ZnO NPs were found to act as natural regulator for plants under stressed and non-stressed conditions by modulating key physiological parameters and thereby enhancing plant growth and development. The changes in lipid peroxidation and antioxidant metabolism of plants have been recorded upon the ZnO NPs supply, which could reduce the oxidative stress and protect plants from damaging effects of ROS. The small size of ZnO-NPs allows its entry in plant cells, assisting in seed germination and growth [34].

SOD is a key antioxidant enzyme [35], in the chloroplast, cytosol, and mitochondria catalyzing the dismutation of superoxide radicals (O_2_−) to H_2_O_2_ and O_2_, thus playing a major role in counteracting oxidative stress [36]. SOD activity was higher in the highest dose in maize [37]. Increased activity of SOD and POX in *Gossypium* and subsequent decrease in lipid peroxidation was reported [38]. Apart from SOD, other enzymes of antioxidant system, such as CAT, GPX, and APX, reduce H_2_O_2_ into water and oxygen in cytoplasm and various cellular organelles [39]. In conditions of stress, induced by NPs [40], oxidative stress has been observed, when the equilibrium between ROS production and the defense system in plant is impaired [41].

Zinc is present in the cystolic and chloroplastic Cu/Zn-SOD enzymes [42,43], which play a critical role against the oxidative stress. Du et al. [44] highlighted the potential benefits of supplementing the Zn NPs as a fertilizer because Zn deficiency in plants is the major concern globally [45]. Dimpka et al. [46] showed that higher concentration of ZnO NPs may also cause the toxicity to the plants. The effects of ZnO NPs in crop plants, such as rice and barley, have been studied and positive influences on cellular redox state, antioxidant defense and growth of plants have been observed [47,48,49]. Prasad et al. [50] reported increased seed germination, along with enhanced seedling vigour in peanut seeds, when treated with 1000 mg/kg of ZnO NPs (average size ~25 nm). The positive effects of ZnO NPs were also seen in early flowering, increased leaf chlorophyll content, increased stem and root growth, and higher pod yield per plant. In addition, Dimpka et al. [51] showed the beneficial effect of ZnO NPs on shoot and root length, plant height, biomass, chlorophyll, grain yield, and uptake in wheat crops, with 40% field moisture capacity. ZnO NPs showed greater positive effects, as compared to ZnO bulk treatment [51].

## 4. Conclusions

This study indicated that ZnO NPs at low doses can act as a seed priming agent, in order to achieve better germination and seedling growth. Germination of maize and wheat is not adversely affected by ZnO NPs. The application of ZnO NPs showed an increase in amylase activity; however, dehydrogenase activity decreased at higher ZnO NPs doses during germination in both maize and wheat. Further root and shoot length, as well as chlorophyll and carotenoid content, increased in the seedlings. In addition, the oxidative stress, induced by the ZnO NPs treatment, was found to alter the antioxidant enzyme composition of both plants. Thus, ZnO NPs can be utilized as a seed priming agent and potential fertilizer for enhancing crop yields in the future. However, the response of plants to ZnO NPs was found to be dose-dependent. Therefore, future studies need to analyze various doses of ZnO NPs (in lab and field conditions), plants (in control conditions), and the presence of different stress conditions, in order to find the optimum doses of ZnO NPs for normal, as well as stressed, environments. The proper use of ZnO NPs would also be beneficial in augmenting crop produce quality for human nutrition.

## Data Availability

Not applicable.
